# Clinical and pathological characteristics, treatment outcome and prognostic factors in adult rhabdomyosarcoma: a monocentric retrospective study

**DOI:** 10.11604/pamj.2022.41.246.31425

**Published:** 2022-03-25

**Authors:** Myriam Saadi, Feryel Letaief, Azza Gabsi, Amina Mokrani, Khedija Meddeb, Amel Mezlini

**Affiliations:** 1 Medical Oncology Department, Institut Salah Azaïz, Tunis, Tunisia

**Keywords:** Adult, rhabdomyosarcoma, prognosis

## Abstract

Rhabdomyosarcoma (RMS) is rare in adults. Our study is the first in Tunisia to report outcomes of adult RMS. We retrospectively analyzed clinical data of adult RMS patients. We collected data regarding clinical characteristics, treatment outcome and prognostic factors. Survival was assessed using the Kaplan Meier method. Forty-seven patients were included. Median age was 39. Twenty-five patients were young adults (53%). Sex ratio (M/F) was 1.9. RMS was localized in 33 patients (70%) and metastatic in 14 patients (30%). Extremities were the most frequent tumor site (40%) followed by trunk (23%). Median tumor size was 9 cm. Pleomorphic RMS was the major subtype (36%). Twenty seven of 33 patients with localized RMS underwent surgery (82%). Relapse free survival (RFS) was 38%. Young adults had a significantly worse RFS than adults aged ≥40 (p = 0.045). Surgery was associated with a significantly better RFS (p = 0.023). Five year overall survival (OS) was 35% and 27% in localized and metastatic RMS respectively. RMS localized in the extremities had significantly poorer OS (p = 0.041), same as non-operated patients (p = 0.025). OS for metastatic RMS was significantly worse after surgery of the primary tumor (p = 0.002). In multivariate analysis, surgery (HR = 0.108; 95%CI (0.023-0.519); p = 0.005) and non-extremity localization (HR = 0.238; 95%CI (0.075-0.751); p = 0.014) were independent prognostic factors for OS in localized RMS. Adults with RMS have poor 5 year OS. Surgery and non-extremity localization were independent prognostic factors for OS in localized RMS.

## Introduction

Rhabdomyosarcoma (RMS) is a very rare disease occurring mostly in children. Its prognosis has been improved in the last thirty years thanks to multimodality treatment [1]. RMS is occasionally seen in adults. It represents less than 1% of adult cancers and less than 4% of adults soft tissue sarcomas (STS) [1]. The management of these tumors in adults is still challenging and data published in this setting is limited [2]. Recent studies suggested that applying pediatric protocols can improve survival [3,4]. Prognostic factors in pediatric RMS are well identified, however, in adult patients, it is still difficult to determine if prognostic factors are similar to those in children [5]. The aim of our study was to describe clinical and pathological features as well as outcome, and to determine prognostic factors of adult RMS treated in a reference center.

## Methods

We conducted a retrospective study including adult RMS patients treated between 1994 and 2017 in Salah Azaiez Institute of Tunisia. The criteria for inclusion were: 1) Age ≥18 years; 2) a histologically-proven RMS: pleomorphic RMS (PRMS), embryonal RMS (ERMS), alveolar RMS (ARMS), mix (ERMS + ARMS) and not otherwise specified (NOS) according to the World Health Organization classification of 2013; 3) patients addressed after surgery and/or chemotherapy outside of our institute or at progression were included. Medical records were reviewed retrospectively including: age, sex, symptoms at presentation, consulting delay, size and site of the primary tumor, imaging data, type of surgery and resection margins (R0 microscopically negative margins, R1 microscopically positive margins and R2 macroscopically positive margins), characteristics of radiotherapy (RT): neoadjuvant, adjuvant or palliative and dose administrated, and chemotherapy (CT): neoadjuvant, adjuvant or palliative plus protocol or regimen administrated with dates of start and end of CT.

Data from medical records was entered into the statistical package for the social sciences (SPSS). Localized RMS and metastatic RMS were analyzed together for epidemiological and clinical characteristics and separately for survival and prognostic factors. Correlation was analyzed using Pearson method. Median follow-up was calculated using the inverse Kaplan Meier method. Overall survival (OS) was calculated as the time between diagnosis and death or date of last visit. For patients with localized RMS, relapse free survival (RFS) was calculated as the time between the start of the treatment and the relapse. Survival curves were established using the Kaplan Meier method. Prognostic factors were determined using Log Rank test in univariate analysis. Significant prognostic factors in univariate analysis were qualified to be introduced into multivariate analysis. Multivariate analysis was done using Cox regression method. Chi 2 test was used to determine independence between variables with a p value < 0.05 to indicate significance. All analysis were performed using SPSS 22.

## M Results

Our study included 47 patients. Median age was 39 (19-77). Two age intervals were predominant: 18-28 and 62-72 years old. Twenty-five patients were young adults: <40 years old (53%). Thirty-one were male and 16 females with a sex ratio of 1.9. Median consulting delay was 3.5 months. Most patients presented with a painless mass (64%). Extremities were the most frequent tumor location (40%) followed by trunk (23%), head and neck (19%) and genitourinary (GU) tract (17%). Median tumor size at diagnosis was 9 cm (min 3 cm, max 26 cm). Tumor size was >5 cm in 89% of cases. PRMS was the most frequent subtype accounting for 36% of cases followed by ERMS (26%), NOS (23%), ARMS (13%) and mix (2%). PRMS was significantly associated with the largest tumor sizes (p = 0.034). ERMS was the most frequent subtype in young adults while PRMS was the most frequent in adults aged 40 or more (p = 0.023). PRMS was mostly located in extremities and trunk while ERMS was mostly located in head and neck (HN). RMS was localized in 33 patients (70%) and metastatic in 14 patients (30%). Lungs were the most common metastatic site (54%) followed by lymph nodes (20%), bone (13%) and liver (13%).

**Localized rhabdomyosarcoma:** twenty-seven out of 33 patients with localized RMS underwent surgery (82%). Resection was R0 in 67% of them, R1 in 18% and R2 in 15%. Four patients received neoadjuvant CT (NACT). All of these patients had head and neck (HN) localization. CT regimens were ifosfamide, vincristine, actinomycine (IVA) for 2 patients and adriamycine, ifosfamide (AI) for 2 patients with a median number of cycle of 3 (1-6). After NACT, 1 patient didn´t show up after 1 cycle and the 3 others achieved partial response after two, 3 and 6 cycles and received radiation therapy (RT). None of them had surgery. Six out of 33 patients with localized RMS received adjuvant CT (18%) with a median number of cycles of 3 (2-6). CT regimens were AI for 3 patients, ifosfamide, vincristine, actinomycine, doxorubicine (IVADO) for 1 patient and PEV (cisplatin, epirubicin, etoposid (VP16)) for 2 patients. Five patients achieved complete response (83%) and one patient had progressive disease. Sixteen patients have been irradiated. RT followed surgery in 12 cases (36%); after R0 resection in 6 cases and R1 or R2 in 6 cases, and followed NACT in 3 cases (9%). RT was symptomatic for a supra vena cava syndrome in one case. Complete response after initial treatment was achieved in 26 out of 33 patients with localized RMS (79%) (Table 1).

**Table 1:** treatment modalities in localized RMS

Treatment	Response to treatment			Total
	CR	PR	PD	
Surgery alone	10	0	1	11
Surgery + RT	9	0	1	10
Surgery + CT + RT	3	0	0	3
Surgery + CT	2	0	1	3
CT + RT	2	1	0	3
CT alone	0	0	0	1 (lost to follow up)
RT alone	0	1	0	1
No treatment	-	-	-	1
Total	26	2	3	33

CT: chemotherapy; RT: radiotherapy; PR: partial response; CR: complete response; PD: progressive disease

Eighteen patients (55%) relapsed in a median time of 5 months. Nine patients presented local recurrence (50%), six presented distant recurrence (33%) and 3 presented local and distant recurrence (17%). Distant recurrence mostly occurred in lungs (56%). Sixteen patients were treated for disease recurrence (89%). CT was the main treatment (13 patients: 81%), administrated alone in 6 cases, with surgery in 4 cases, with RT in 1 case, with both RT and surgery in 2 cases. Most used CT regimens were AI (4 patients: 31%), PAI (cisplatin, doxorubicine, ifosfamide) (2 patients: 15%) and mesna, doxorubicin, ifosfamide, and dacarbazine (MAID) (2 patients: 15%). Other regimens used in 1 case each were IVA, VAC-VAD (vincristine, actinomycin, cyclophosphamide - vincristine, adriamycin), VIP (etoposide (VP16), ifosfamid, cisplatin), EP (epirubicine, ciplatine) and CD (cisplatine, dacarbazine). Eight patients underwent surgery after recurrence (44%). Resection was complete in 6 cases with local recurrence and palliative in 2 cases. Four patients had RT after recurrence (22%). RT concerned metastatic symptomatic sites in 3 cases and was adjuvant after surgery of a local recurrence in 1 case.

**Metastatic RMS:** fourteen patients had metastatic RMS at diagnosis. All but one received CT. Median number of cycles was 3 (1-7). Main CT regimens were IVA and AI, prescribed in 5 (36%) and 3 cases (21%) respectively. Other regimens used for 1 case each were VAC, PAI, MAID and CYVADIC (cyclophosphamid, vincristin, adriamycin, dacarbazine). After 1st line CT, one patient achieved complete response (7%), 4 had partial response (29%) and 8 had progressive disease (57%). Four patients received second line CT with AI, VIP, oral cyclophosphamide and irinotecan-cisplatin. One patient had stable disease, one had progressive disease and 2 patients died. No one received third line CT. Six out of 14 patients with metastatic RMS had surgery (43%). Four patients received radiation therapy (28%), following response to CT in 2 cases and palliative in 2 cases. Table 2 shows response to different CT regimens.

**Table 2:** response to chemotherapy for patients with metastatic RMS

CT line	CT regimen	Number of patients according to response to CT			
		CR	PR	SD	PD
First line	IVA	0	1	0	4
	AI	0	1	0	3
	VAC	0	0	0	1
	PAI	0	1	0	0
	MAID	0	1	0	0
	CYVADIC	1	0	0	0
Second line	AI	0	0	1	0
	VIP	0	0	0	1
	Endoxan	0	0	0	1
	Irinotecan-cisplatine	0	0	0	1

CT: chemotherapy; CR: complete response; PR: partial response; SD: stable disease; PD: progressive disease; IVA: ifosfamide, vincristine, actinomycine; AI: adriamycine, ifosfamide; VAC: vincristine, actinomycin, cyclophosphamide; PAI: cisplatin, doxorubicine, ifosfamide; MAID: mesna, doxorubicin, ifosfamide, and dacarbazine; CYVADIC: cyclophosphamid, vincristin, adriamycin, dacarbazine

**Figure 1:**
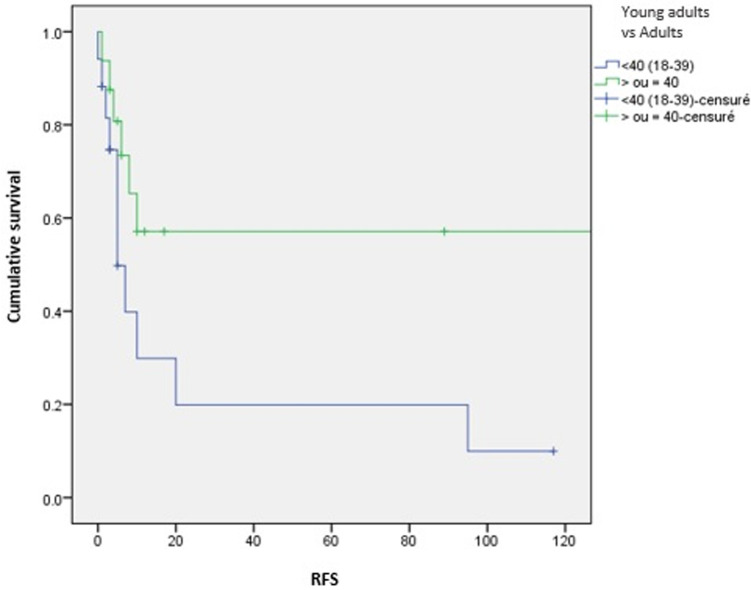
relapse free survival according to age

**Figure 2:**
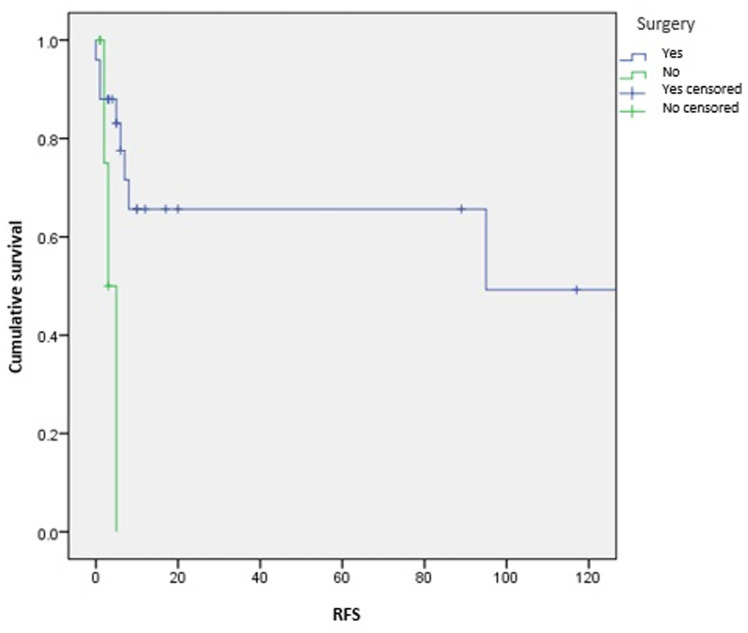
relapse free survival according to surgery

**Figure 3:**
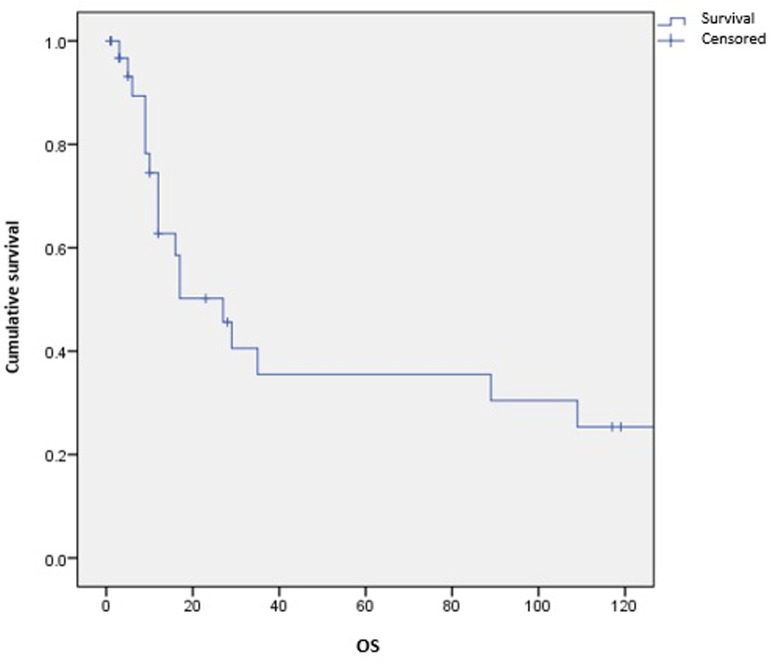
overall survival for localized RMS

**Figure 4:**
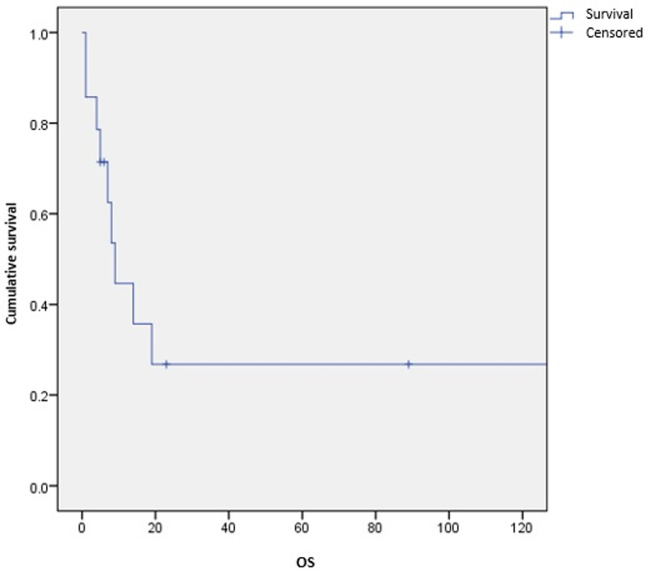
overall survival for metastatic RMS

**Figure 5:**
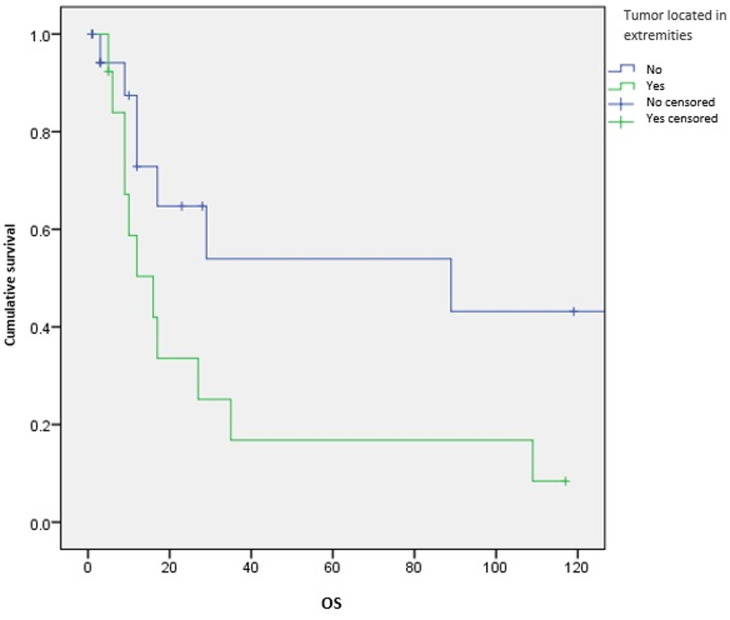
overall survival according to tumor localization in localized RMS

**Figure 6:**
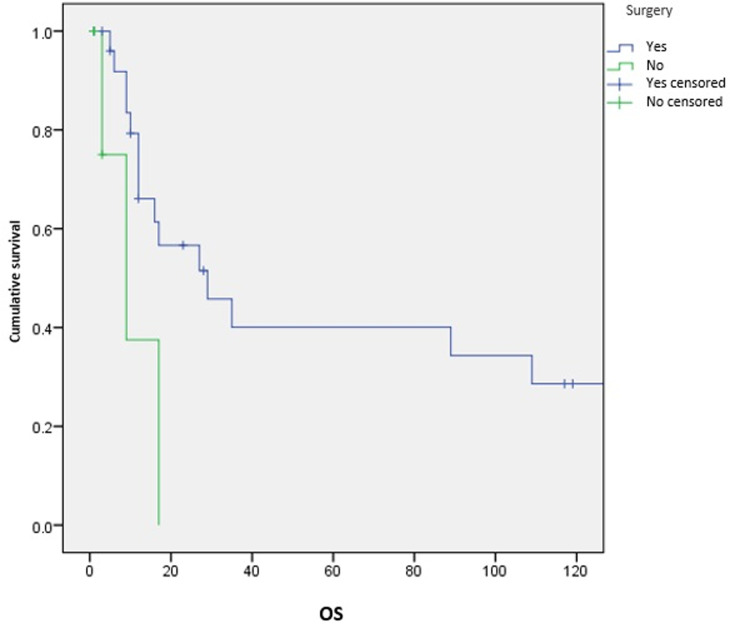
overall survival according to surgery in localized RMS

**Survival and prognostic factors:** median RFS for localized RMS was 5 months. Five-year RFS was 38%. Adults aged 40 or older had significantly better RFS than young adults (p = 0.045) (Figure 1). Surgery was also significantly associated with a better RFS (p = 0.023) (Figure 2). In multivariate analysis, none of these prognostic factors reached statistical significance. Median OS was 12 months and 7.5 months for localized and metastatic RMS respectively. Five year OS was 35% for localized RMS and 27% for metastatic RMS (Figure 3, Figure 4). In localized RMS, RMS of the extremities and non-operated patients had significantly poorer OS with p = 0.041 (Figure 5) and p = 0.025 (Figure 6) respectively. In multivariate analysis, surgery (HR = 0.108; 95%CI (0.023-0.519); p = 0.005) and non-extremity localization (HR = 0.238; 95%CI (0.075-0.751); p = 0.014) were independent prognostic factors for OS in localized RMS (Table 3, Table 4).

**Table 3:** univariate analysis of prognostic factors

Localized rhabdomyosarcoma (RMS)		
Prognostic factors	p (OS)	p (RFS)
Sex	0.942	0 .799
Age	0.870	0.045
Size	0.628	0.147
Histologic subtype	0.193	0.612
Site	0.041	0.804
Node extension (N)	0.202	0.482
Surgery	0.025	0.023
Margins	0.513	0.505
Radiotherapy	0.964	0.994
Chemotherapy	0.914	0.208
Relapse	0.722	Non applicable
**Metastatic RMS**		
**Prognostic factors**	**p (OS)**	**p (RFS)**
Surgery of the primary	0.002	Non applicable
Chemotherapy	0.014	Non applicable

**Table 4:** multivariate analysis of prognostic factors

Rhabdomyosarcoma (RMS)			
**Prognostic factors**	**p**	**HR**	**CI (95%)**
Age	0.077	2.544	0.903-7.169
Surgery	0.755	0.770	0.148-3.989
**OS for localized RMS**			
**Prognostic factors**	**p**	**HR**	**CI (95%)**
Site	0.014	0.238	0.075-0.751
Surgery	0.005	0.108	0.023-0.519
**OS for metastatic RMS**			
**Prognostic factors**	**p**	**HR**	**CI (95%)**
Surgery of the primary	0.947	0.315	0.144-1.074
Chemotherapy	0.327	0.204	0.007-5.663

HR: hazard ratio; OS: overall survival

**Figure 7:**
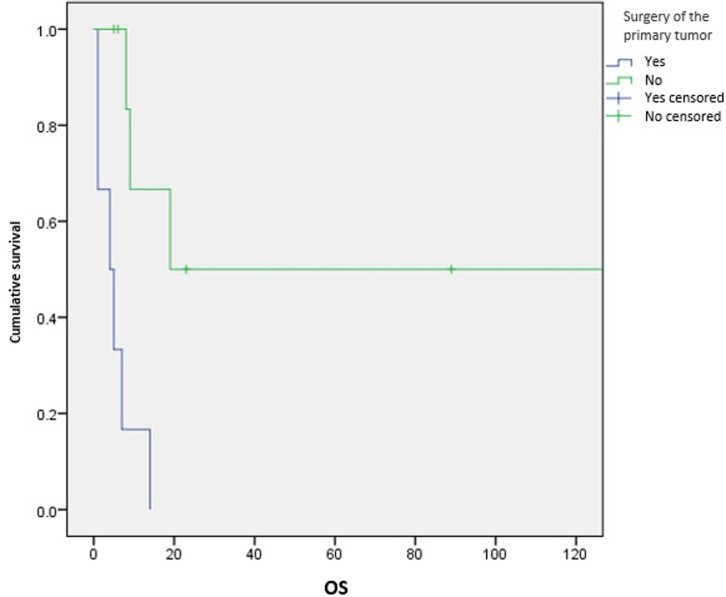
overall survival according to surgery of the primary tumor in metastatic RMS

OS for metastatic RMS was significantly worse after surgery of the primary tumor (p = 0.002) (Figure 7). Five year OS was 20% for metastatic PRMS and 50% for non PRMS subtypes but the difference wasn´t statistically significant (p = 0.581). No prognostic factor reached statistical significance for OS in metastatic RMS in multivariate analysis.

## Discussion

Our study describes clinical characteristics, outcomes and prognostic factors of 47 adult patients with RMS treated at a single reference center. Median age was 39. Extremities were the most common localization (19 cases, 40%). Main clinical presentation was a non-symptomatic mass. Size was >5 cm in 89% of cases. RMS was localized in 33 cases (70%). PRMS was the most frequent subtype, reported in 36% of cases, followed by ERMS (26%), NOS (23%), ARMS (13%) and mix (2%). In localized RMS, we report 18 recurrences after a median follow up of 35 months. Five year RFS was 38%. Five year OS was 35% for localized RMS and 27% for metastatic RMS.

There are no specific guidelines for the management of adult RMS. Most published studies are retrospective, and randomized trials are difficult to conduct with such a low incidence. To our knowledge, our study seems to be the first in Tunisia and North Africa to report outcomes of adult RMS in the region. It´s a challenge for every oncologist to deal with adult RMS. The question is whether to treat it as an adult STS or to extrapolate data from pediatric guidelines. Authors are now subdividing treatment approaches according to histologic subtype. PRMS, a subtype almost exclusively seen in adults, seems to be more aggressive and less chemo-sensitive than other subtypes. Published data suggests that PRMS should be treated according to adult STS guidelines. While ERMS and ARMS should be treated according to pediatric guidelines [5,6].

Our results are consistent with those reported in the literature. The majority of our patients had PRMS and were treated as adult STS according to published recommendations [3,7,8]. Adult RMS occurs more frequently in man with a sex ratio of 1.75 [5]. We report a sex ratio of 1.9. Median age in our study is 39 and 37 in published studies [5]. Authors report a median delay to consult of 3 months [9,10] which is the same in our study (3.5 months). Main presentation is an asymptomatic mass in our study as well as in previous studies [11]. Median tumor size at diagnosis is 8 cm in the literature [5] and 9 cm in our study. Extremities are the most common site reported in literature [5,8,11] and in our study. More than 20% of RMS are metastatic at diagnosis, lungs being the most frequent site of metastasis [8] which is consistent with our findings. Relapse rates reported in the literature range from 33 to 57% [5] versus 54% in our study.

Relapse occurs most frequently in the first year [5]. Sixty seven per cent (67%) of our patients relapsed during the first year. Five year RFS was 38%, similar to rates reported in other published series [12-14]. We report a 5-year OS of 35% for localized RMS, notably inferior to literature rates that ranges from 43 to 52% [5,6,12-20]. Median OS was 12 months for localized RMS, consistent with only one retrospective study of 45 cases [20] and notably inferior to the majority of other studies where median OS ranges from 35 to 45 months [5,6,13-15], probably due to the fact that very few patients received CT as an adjuvant treatment in our study. CT was in fact mostly prescribed at disease recurrence and for metastatic RMS. Patients with metastatic RMS had a 5-year OS rate of 27% similar to some studies [13,16] and a little superior to other ones [5,6,14,16,17,19] as almost all our patients with metastatic RMS received CT. Independent prognostic factors reported in our study were tumor site and surgery for OS in localized RMS. Both have been reported in several studies [5,12,15,18].

## Conclusion

Adult patients with RMS have poor 5-year overall survival. Surgery and non-extremity localization were independent prognostic factors for OS in localized disease. No prognostic factor reached statistical significance for metastatic disease in multivariate analysis. Management of adult RMS, a rare and aggressive tumor, requires a combination of surgery, chemotherapy and radiotherapy. Collaboration between pediatric and adult oncologists is essential to develop research and improve the outcome of adult RMS.

### What is known about this topic

- There are no specific guidelines for adult RMS;
- Pleomorphic RMS is almost exclusively seen in adults and is commonly be treated as adult soft tissue sarcomas;
- Embryonal and alveolar RMS are commonly treated according to pediatric guidelines.

### What this study adds

- The prognosis of adult rhabdomyosarcoma is still poor;
- Adults aged 40 or older had significantly better relapse-free-survival than young adults;
- Surgery and tumor site are independent prognostic factors for overall survival in localized RMS.
